# *MdVQ12* confers resistance to *Valsa mali* by regulating *MdHDA19* expression in apple

**DOI:** 10.1111/mpp.13411

**Published:** 2023-12-10

**Authors:** Pengliang Han, Ruotong Zhang, Rui Li, Fudong Li, Jiajun Nie, Ming Xu, Chengli Wang, Lili Huang

**Affiliations:** ^1^ State Key Laboratory for Crop Stress Resistance and High‐Efficiency Production, College of Plant Protection Northwest A&F University Yangling China

**Keywords:** apple Valsa canker, genetic transformation, *Malus* × *domestica*, plant immunity, transcription factor

## Abstract

Valine‐glutamine (VQ) motif‐containing proteins play a crucial role in plant biotic stress responses. Apple Valsa canker, caused by the ascomycete *Valsa mali*, stands as one of the most severe diseases affecting apple trees. Nonetheless, the underlying resistance mechanism of VQ proteins against this disease has remained largely unexplored. This study reports MdVQ12, a VQ motif‐containing protein, as a positive regulator of apple Valsa canker resistance. Genetic transformation experiments demonstrated that *MdVQ12* overexpression increased resistance to *V. mali*, while gene silencing lines exhibited significantly reduced resistance. MdVQ12 interacted with the transcription factor MdWRKY23, which bound to the promoter of the histone deacetylase gene *MdHDA19*, activating its expression. *MdHDA19* enhanced apple resistance to *V. mali* by participating in the jasmonic acid (JA) and ethylene (ET) signalling pathways. Additionally, *MdVQ12* promoted the transcriptional activity of MdWRKY23 towards *MdHDA19*. Our findings reveal that *MdVQ12* enhances apple resistance to *V. mali* by regulating *MdHDA19* expression and thereby regulating the JA and ET signalling pathways, offering potential candidate gene resources for breeding apple Valsa canker‐resistant germplasm.

## Graphical abstract

*MdVQ12* activates *MdHDA19* to enhance apple resistance against *Valsa mali*.

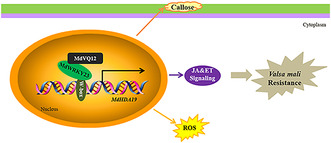

## 1 INTRODUCTION

In the plant immune system, the first line of defence against pathogenic microorganisms, pattern‐triggered immunity (PTI) is mediated by membrane‐localized pattern recognition receptors (PRRs) that recognize microbial‐ or pathogen‐associated molecular patterns (MAMPs or PAMPs). Subsequently, effector‐triggered immunity (ETI) recognizes pathogen effectors by intracellular nucleotide‐binding leucine‐rich repeat receptors (NLRs) to stimulate the immune response (Jones & Dangl, 2006).

When PTI or ETI is triggered, a cascade of signals will expand and extend from the site of invasion to the downstream of immunity, causing plants to limit pathogen colonization and invasion. Transcription factors (TFs), especially WRKYs, play a critical role in this process. For example, in the immune response induced by flg22, there is a mitogen‐activated protein kinases cascade (MAPK), an indispensable component in the process of plant immune signal transduction. The immune signal is transmitted downward through MEKK1, which affects the downstream TFs such as WRKY22 and WRKY29 to achieve immune signal transduction (Asai et al., 2002). MPK3 and MPK6 can directly phosphorylate WRKY33 to promote phytoalexin biosynthesis in *Arabidopsis* (Mao et al., 2011). Phosphorylation of WRKY8 by MAPK functions in the defence response in *Nicotiana benthamiana* (Ishihama et al., 2011). These studies demonstrate that WRKY TFs play an important role in coordinating plant immunity signalling.

WRKY TFs are plant‐specific factors that can control the transcription of various genes and participate in the regulation of a variety of plant life activities. They were first identified in sweet potato by Ishiguro and Nakamura (1994). Subsequently, several WRKY TFs have been reported in more than 20 plant species, including rice, *Arabidopsis*, and tomato (Huang et al., 2012; Wu et al., 2005). WRKY TFs are important regulators of the defence response at the transcriptional level. Knockdown of *CaWRKY1* in pepper resulted in a reduction of *Xanthomonas* growth in leaves, demonstrating its important regulatory role in pathogen‐induced immune responses (Oh et al., 2010). *WRKY11* and *WRKY17* negatively regulate plant resistance to *Pseudomonas syringae* in *Arabidopsis* (Journot‐Catalino et al., 2006). *MdWRKY100* can improve leaf resistance to *Colletotrichum gloeosporioides* in apple (Zhang et al., 2019). However, the contribution and regulatory mechanism through which WRKY TFs are involved in protecting apple from *V. mali* invasion is not yet clear.

TFs often require some transcriptional regulators to assist their function, and VQ motif‐containing proteins are one of the key proteins. VQ proteins play an important role in the response of plants to biotic stress (Jing & Lin, 2015). The transcription level of *AtVQ23*/*SIB1* was strongly induced by *P. syringae* and *Botrytis cinerea* infection, and gene overexpression improved the disease resistance in *Arabidopsis* (Lai et al., 2011; Xie et al., 2010). Overexpression of *AtVQ10* enhanced resistance to *B. cinerea*, whereas the *vq10* mutant reduced resistance to the pathogen (Chen et al., 2018). As transcriptional regulators, VQ proteins can interact with a variety of TFs, including WRKY, to participate in the response to biotic stress (Chi et al., 2013; Jing & Lin, 2015). For instance, AtVQ23 and AtVQ16 could interact and activate WRKY33 by enhancing its DNA‐binding activity to improve plant disease resistance (Lai et al., 2011). Nevertheless, there are few studies on the joint regulatory roles of VQ proteins and WRKY TFs in resistance to *V. mali*.

Apple Valsa canker, caused by the fungus *V. mali*, is one of the most severe diseases of apple (Abe et al., 2007; Xu et al., 2020; Yin et al., 2016). In this study, *MdVQ12* was upregulated during *V. mali* infection. Therefore, we thought that it might be involved in the regulation of resistance to apple Valsa canker. However, the regulatory mechanism of *MdVQ12* to *V. mali* resistance remains a mystery. Here, we reported that *MdVQ12* confers apple resistance to *V. mali* by regulating the expression of the histone deacetylase gene *MdHDA19*. Histone acetylation plays an important role in plant epigenetic modification and this process is reversible, mainly including histone acetyltransferases (HATs) and histone deacetylases (HDACs) (Ma et al., 2013). Histone deacetylases are critical for plant growth, development, and stress response (Ma et al., 2013; Zhou et al., 2005). This study provides new insights into the molecular mechanism of the resistance to *V. mali* and an important reference value for the breeding of apple disease resistance.

## 2 RESULTS

### 2.1 *MdVQ12* positively regulates apple resistance to *V. mali*

The analysis of MdVQ12's conserved domain identified a VQ domain (Figure S1a). Protein feature visualization showed that this protein is located inside the cytomembrane, indicating that it is an intracellular localization protein (Figure S1b). Subcellular localization analysis revealed that it was localized to the nucleus (Figure S1c). Furthermore, upregulation of *MdVQ12* relative expression was detected during *V. mali* inoculation (Figure S1d), suggesting its involvement in the responses to *V. mali* infection. Next, stable transgenic apple calli expressing *MdVQ12* were established to characterize the function of *MdVQ12* (Figure S2a). Calli infected for 4 days were used to determine lesion area, H_2_O_2_, and O^2−^ levels. Results indicated that *MdVQ12*‐OE‐2/3/6 apple calli exhibited increased resistance to *V. mali* infection, with 31.4% ± 5.6%, 32.4% ± 6.3%, and 25.1% ± 6.4% reductions in lesion areas compared to the wild type (WT) (Figure S2b,c). Additionally, *MdVQ12‐*overexpressing apple calli exhibited significantly higher levels of reactive oxygen species (ROS) compared with the WT (Figure S2d,e). We then made an association study by showing the *R* ^2^ and *p* values between levels of *MdVQ12* transcripts and lesion area, H_2_O_2_ content, and O^2−^ content (Table S1). These results demonstrated that *MdVQ12*'s capacity to enhance resistance in apple calli against *V. mali*.

**FIGURE 1:**
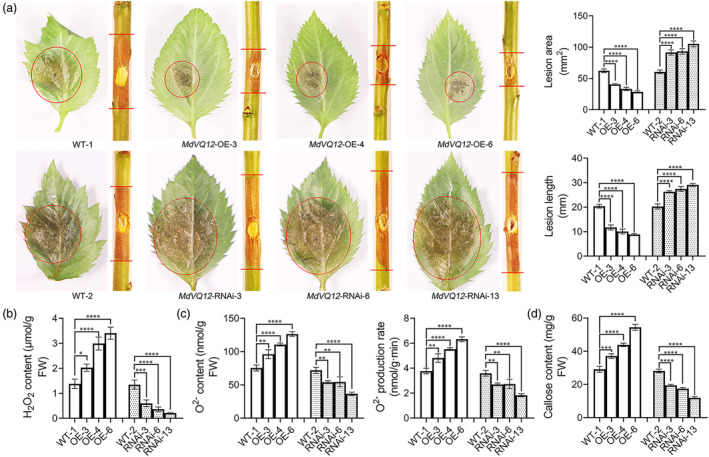
*MdVQ12* positively regulates apple resistance to *Valsa mali*. (a) Disease symptoms and lesion areas/lengths of wild type (WT), *MdVQ12*‐OE, and *MdVQ12*‐RNAi apple leaves and twigs at 36 h post‐inoculation (hpi) and 48 hpi, respectively. (b) H_2_O_2_ contents of WT, *MdVQ12*‐OE, and *MdVQ12*‐RNAi apple leaves at 36 hpi. (c) O^2−^ contents and production rates of WT, *MdVQ12*‐OE, and *MdVQ12*‐RNAi apple leaves at 36 hpi. (d) Callose contents of WT, *MdVQ12*‐OE, and *MdVQ12*‐RNAi apple leaves at 36 hpi. *p < 0.05, **p < 0.01, ***p < 0.001, ****p < 0.0001; *t* test. Data are shown as mean ± *SD*.

Stable transgenic GL‐3 tissue culture seedlings were obtained for further investigating *MdVQ12* functionality. Agarose gel electrophoresis experiments demonstrated the presence of DNA bands exclusively in *MdVQ12*‐OE lines (Figure S3a). Western blot analysis confirmed the detection of MdVQ12‐HA in *MdVQ12*‐overexpression (OE) lines (Figure S3b). Reverse transcription‐quantitative PCR (RT‐qPCR) analysis revealed higher expression of *MdVQ12* in *MdVQ12*‐OE lines compared to the WT (Figure S3c). Similarly, gene silencing lines were characterized. Fluorescent labelling indicated the presence of green fluorescence solely in *MdVQ12*‐RNAi lines (Figure S4a). Agarose gel electrophoresis experiments detected DNA bands specifically in *MdVQ12*‐RNAi lines (Figure S4b). RT‐qPCR analysis showed lower expression of *MdVQ12* in *MdVQ12*‐RNAi lines compared with the WT (Figure S4c). Subsequently, they underwent *V. mali* infection, with leaves and twigs exposed for 36 and 48 h, respectively. ROS and callose contents and the O^2−^ production rate of the leaves were measured at 36 h post‐inoculation (hpi). The results showed that *MdVQ12*‐OE‐3/4/6 led to enhanced resistance in apple leaves and twigs against *V. mali*. Lesion areas and lengths were reduced by approximately 35.1% ± 4.2%, 46.7% ± 5%, and 54.3% ± 4% and 42.5% ± 3.4%, 50.7% ± 4.8%, and 56.6% ± 2.1%, respectively, relative to the WT. Conversely, *MdVQ12*‐RNAi‐3/6/13 apple leaves and twigs exhibited larger and longer lesions than the WT. Lesion areas and lengths were increased by approximately 51.4% ± 9.7%, 54.4% ± 15.3%, and 74% ± 1.6% and 29.9% ± 7.5%, 35.4% ± 4.2%, and 43.6% ± 9.5%, respectively, relative to the WT (Figure 1a). Moreover, *MdVQ12*‐overexpressing GL‐3 lines exhibited higher ROS and callose contents, as well as a greater O^2−^ production rate than the WT, while gene silencing lines showed lower values compared to the WT. The H_2_O_2_ content, O^2−^ content, O^2−^ production rate, and callose content of *MdVQ12*‐OE‐3/4/6 were 1.49 ± 0.29, 2.18 ± 0.13, and 2.5 ± 0.36 and 1.28 ± 0.09, 1.47 ± 0.11, and 1.68 ± 0.12 and 1.28 ± 0.09, 1.47 ± 0.11, and 1.68 ± 0.12 and 1.28 ± 0.04, 1.51 ± 0.13, and 1.87 ± 0.12 times higher than the WT, respectively. In contrast, they were reduced by 55.7% ± 6.9%, 73.3% ± 4.6%, and 84.2% ± 2.8% and 24.7% ± 4.3%, 24.2% ± 8.1%, and 48.9% ± 4.5% and 24.7% ± 4.3%, 24.2% ± 8.1%, and 48.9% ± 4.5% and 30.9% ± 4.6%, 37.8% ± 4.9%, and 57.5% ± 4% in *MdVQ12*‐RNAi‐3/6/13, respectively, relative to the WT (Figure 1b–d), suggesting that *MdVQ12* enhances GL‐3 seedling resistance to *V. mali*. We then made an association study by showing the *R* ^2^ and *p* values between levels of *MdVQ12* transcripts and each trait (i.e., lesion area, lesion length, H_2_O_2_ content, O^2−^ content, O^2−^ production rate, and callose content) (Table S1). The previous results confirm that *MdVQ12* positively regulates apple resistance against *V. mali*.

### 2.2 *MdVQ12* *‐*induced resistance is *MdWRKY23*‐dependent

**FIGURE 2:**
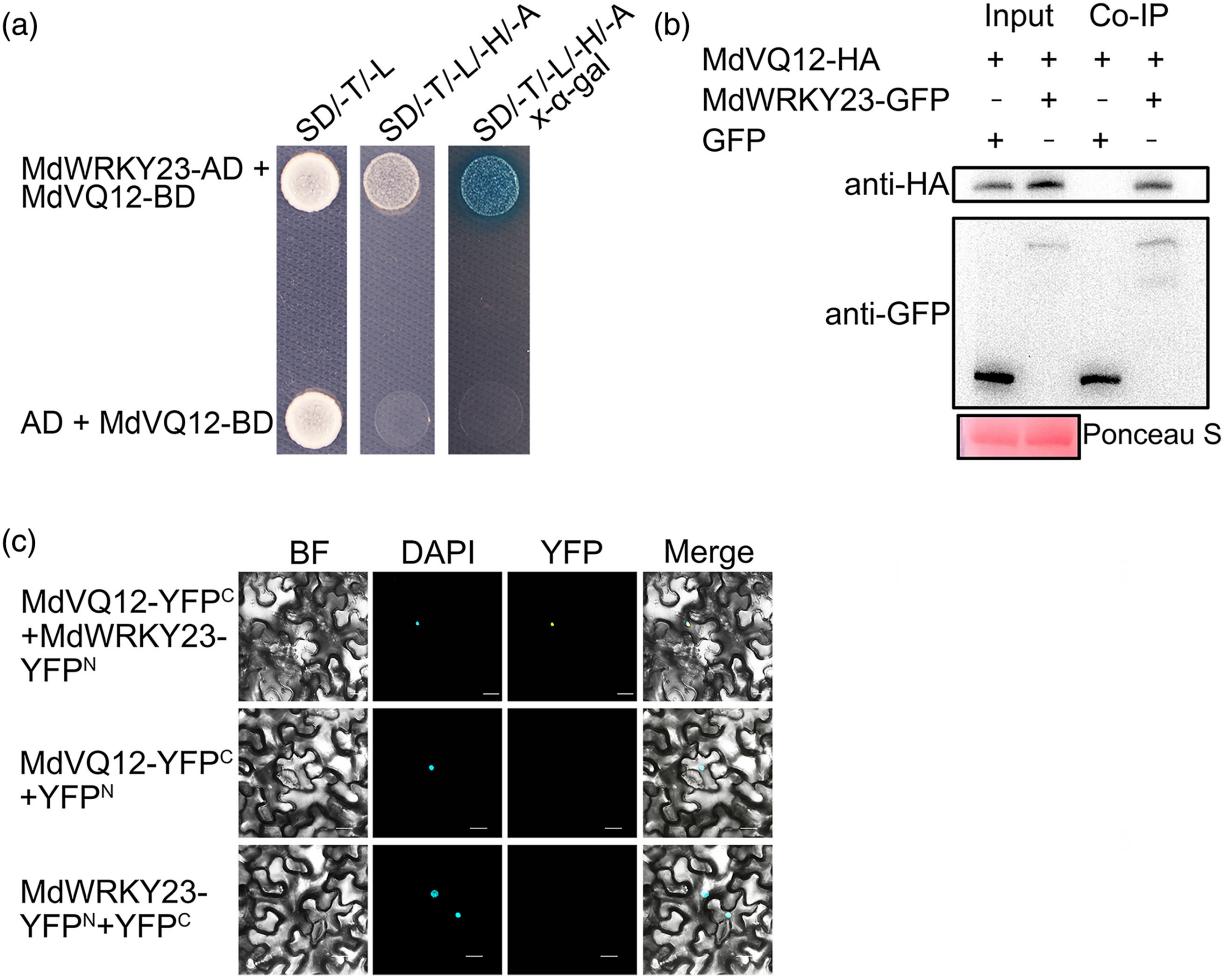
MdVQ12 can interact with MdWRKY23. (a) MdVQ12 can interact with MdWRKY23 in yeast two‐hybrid (Y2H) assays. (b) MdVQ12 can interact with MdWRKY23 in co‐immunoprecipitation (Co‐IP) assays. (c) MdVQ12 can interact with MdWRKY23 in bimolecular fluorescence complementation (BiFC) assays. Bars = 20 μm.

VQ proteins modulate plant disease resistance by interacting with TFs to regulate their transcriptional activities (Jing & Lin, 2015; Li et al., 2014). Therefore, to explore the molecular mechanism of *MdVQ12* in *V. mali* resistance, we employed immunoprecipitation‐mass spectrometry (IP‐MS) to identify interacting TFs. Among the candidate proteins, the WRKY TF MdWRKY23 demonstrated interaction with MdVQ12 in yeast two‐hybrid (Y2H) assays (Figure 2a). This interaction was further confirmed through co‐immunoprecipitation (Co‐IP) and bimolecular fluorescence complementation (BiFC) assays. Co‐IP assay results showed MdVQ12‐HA detection only in the presence of MdWRKY23‐GFP (Figure 2b). Additionally, the BiFC assay revealed fluorescence when MdVQ12 and MdWRKY23 were co‐expressed, confirming their interaction (Figure 2c). These results establish that MdVQ12 interacts with MdWRKY23.

In the study, MdWRKY23 was divided into seven fragments, and the VQ domain was removed from MdVQ12 to assess the necessity of WRKY and VQ domains for the interaction between MdWRKY23 and MdVQ12 via Y2H assays. Results indicate that the WRKY domain combining the N‐terminal segment can interact with MdVQ12 (Table S2). This underscores the essential role of the WRKY domain in facilitating the MdWRKY23‐MdVQ12 interaction.

To investigate the relationship between *MdVQ12* and *MdWRKY23* in *V. mali* resistance, *MdWRKY23* was silenced while overexpressing *MdVQ12* in GL‐3 tissue culture seedling leaves. Interestingly, the enhanced resistance to *V. mali* conferred by *MdVQ12* alone was lost after *MdWRKY23* silencing (Figure S5), indicating that *MdVQ12*'s ability to enhance apple resistance is contingent upon the presence of *MdWRKY23*.

### 2.3 MdWRKY23 can bind to the *MdHDA19* promoter to activate its expression

**FIGURE 3:**
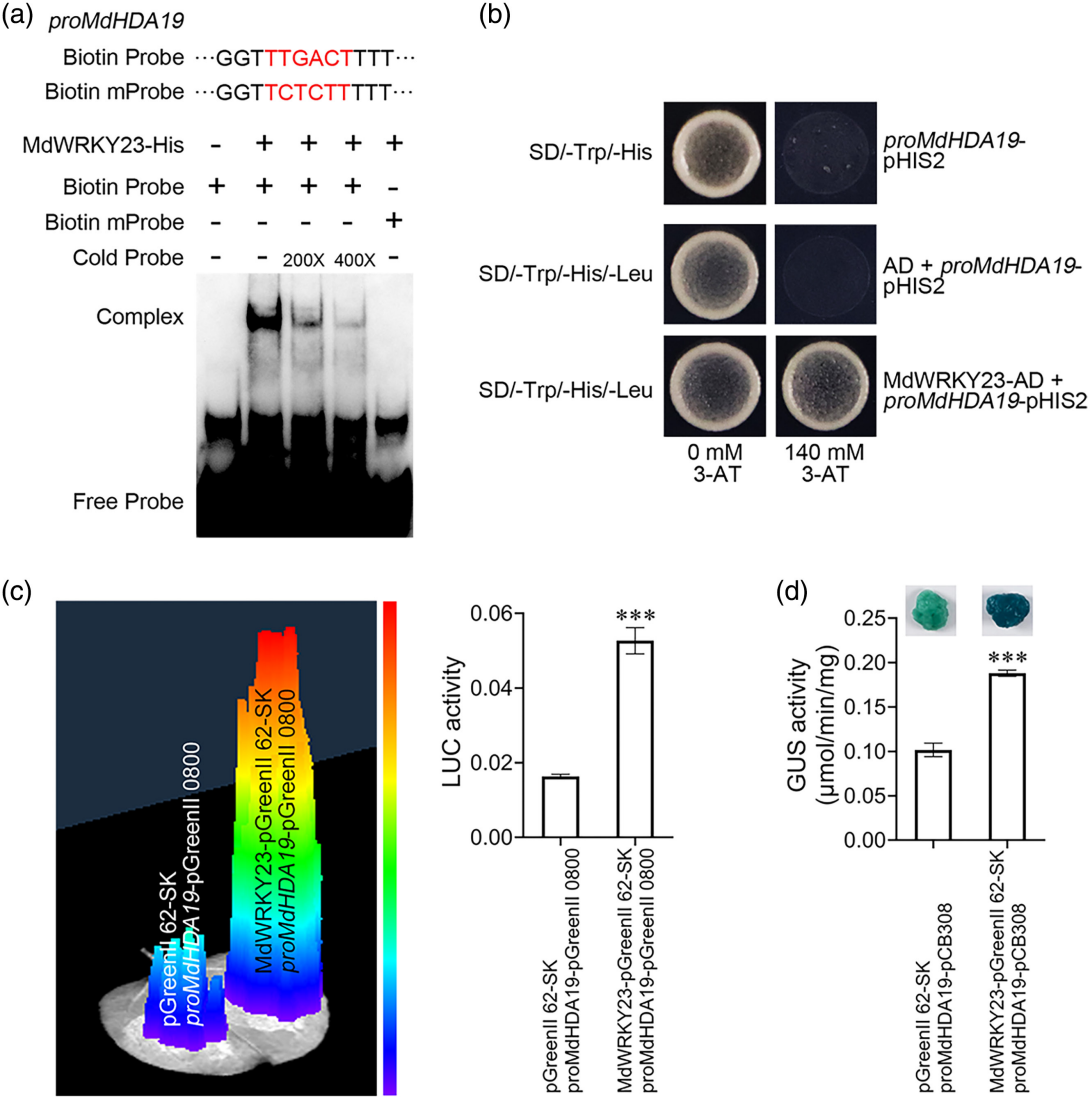
MdWRKY23 can bind to the *MdHDA19* promoter to activate its expression. (a) MdWRKY23 can bind to the *MdHDA19* promoter in electrophoretic mobility shift (EMSA) assays. (b) MdWRKY23 can bind to the *MdHDA19* promoter in yeast one‐hybrid (Y1H) assays. (c) MdWRKY23 can activate *MdHDA19* expression in luciferase (LUC) imaging and LUC activity assays. (d) MdWRKY23 can activate *MdHDA19* expression in β‐glucuronidase (GUS) staining and GUS activity assays. ***p < 0.001; *t* test. Data are shown as mean ± *SD*.

DNA affinity purification sequencing (DAP‐seq) identified genes bound by MdWRKY23, elucidating its role in regulating resistance against *V. mali*. *MdHDA19* (a histone deacetylase gene) emerged as a downstream target, and was confirmed via electrophoretic mobility shift assay (EMSA) and yeast one‐hybrid (Y1H) assays (Figure 3a,b).

To uncover the transcriptional regulation of *MdHDA19* by MdWRKY23, we co‐infiltrated *N. benthamiana* leaves with constructs pGreenII 62‐SK + *proMdHDA19*‐pGreenII 0800 (combination 1) and MdWRKY23‐pGreenII 62‐SK + *proMdHDA19*‐pGreenII 0800 (combination 2). The luminescence signal and luciferase (LUC) activity were weaker in the leaves of combination 1 than in the leaves of combination 2 (Figure 3c). Subsequently, apple calli and *N. benthamiana* leaves were co‐infiltrated with pGreenII 62‐SK + *proMdHDA19*‐pCB308 (combination 3) and MdWRKY23‐pGreenII 62‐SK + *proMdHDA19*‐pCB308 (combination 4). β‐glucuronidase (GUS) staining intensity and GUS activity mirrored luminescence signal and LUC activity (Figure 3d), suggesting that MdWRKY23 can transcriptionally activate *MdHDA19* expression.

### 2.4 *MdHDA19* positively modulates apple resistance to *V. mali*

Histone acetylation is regulated by histone acetyltransferases and deacetylases, which crucially regulates gene expression. *HDA19* has been reported to positively modulate the resistance to the fungal pathogen *Alternaria* in *Arabidopsis* by regulating several ethylene (ET) and jasmonic acid (JA) signal transduction‐related genes to participate in the ET and JA signalling pathways (Zhou et al., 2005). Therefore, we hypothesized that *MdHDA19* similarly contributes to apple Valsa canker resistance. To explore the function of *MdHDA19*, we conducted transient expression assays. The transgenic leaves of GL‐3 tissue culture seedlings of *MdHDA19* and empty vectors (EVs) were generated (Figure S6a,c) and infected with *V. mali* for 32 h. Results confirmed that *MdHDA19*‐OE‐1/5 apple leaves exhibited enhanced resistance to *V. mali*, with lesion areas decreasing by 42.2% ± 3.9% and 54.4% ± 2.2% compared to EV. Conversely, lesion areas in *MdHDA19*‐RNAi‐2/5 apple leaves were 64.4% ± 14.1% and 55% ± 11.7% larger than EV (Figure S6b,d). In addition, we made an association study by showing the *R* ^2^ and *p* values between levels of *MdHDA19* transcripts and lesion area (Table S1). These results demonstrated that *MdHDA19* bolsters GL‐3 leaf resistance against *V. mali*.

To explore whether *MdHDA19* is involved in ET and JA signalling pathways, we measured the relative expression of ET (*MdERF1*) and JA (*MdCOI1*, *MdMYC2*, *MdLOX3*, and *MdVSP2*) signal transduction‐related genes in apple leaves. The results showed that they were all upregulated when *MdHDA19* was overexpressed compared with the EVs (Figure S7).

Because MdHDA19 is a histone deacetylase, we investigated whether it has histone deacetylase activity and whether the activation of this activity is related to the ET and JA pathways. In apple leaves overexpressing *MdHDA19*, we quantitatively measured the expression levels of genes related to the ET and JA pathways after treatment with the histone deacetylase inhibitor trichostatin A (TSA). The results demonstrated that after TSA treatment, the expression levels of related genes were significantly decreased in the apple leaves overexpressing *MdHDA19*, with some even decreasing to levels close to the control (Figure S8). These findings indicate that MdHDA19 possesses histone deacetylase activity and its activation is associated with both ET and JA signalling pathways.

**FIGURE 4:**
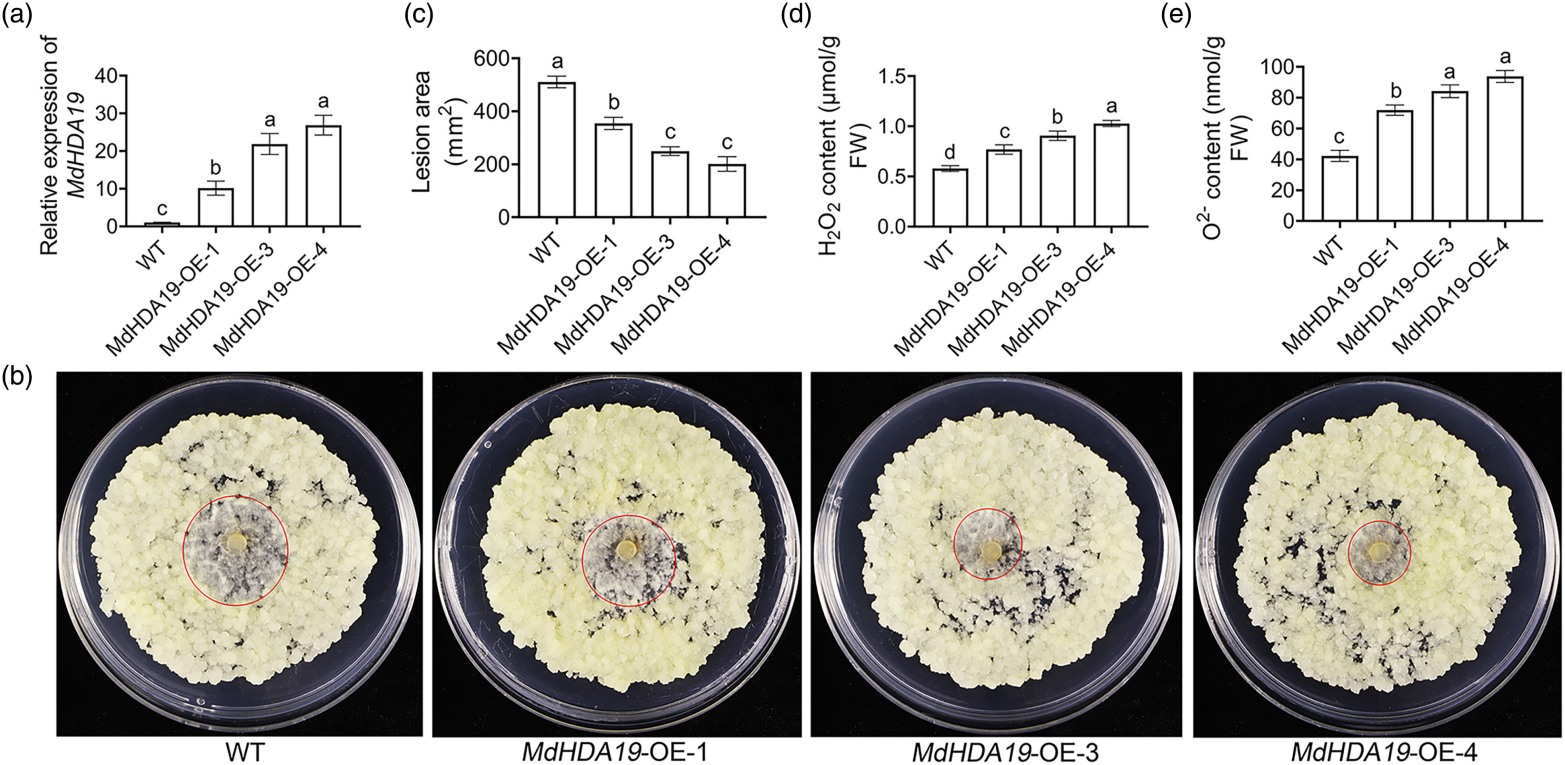
*MdHDA19* confers apple calli resistance to *Valsa mali*. (a) Relative expression of *MdHDA19*. (b) Disease symptoms of wild‐type (WT) and *MdHDA19*‐OE apple calli at 3 days post‐inoculation (dpi). (c) Lesion areas of WT and *MdHDA19*‐OE apple calli at 3 dpi. (d) H_2_O_2_ contents of WT and *MdHDA19*‐OE apple calli at 3 dpi. (e) O^2−^ contents of WT and *MdHDA19*‐OE apple calli at 3 dpi. Bars with different letters are significantly different at *p* < 0.05 according to one‐way analysis of variance (Tukey's test). Data are shown as mean ± *SD*.

**FIGURE 5:**
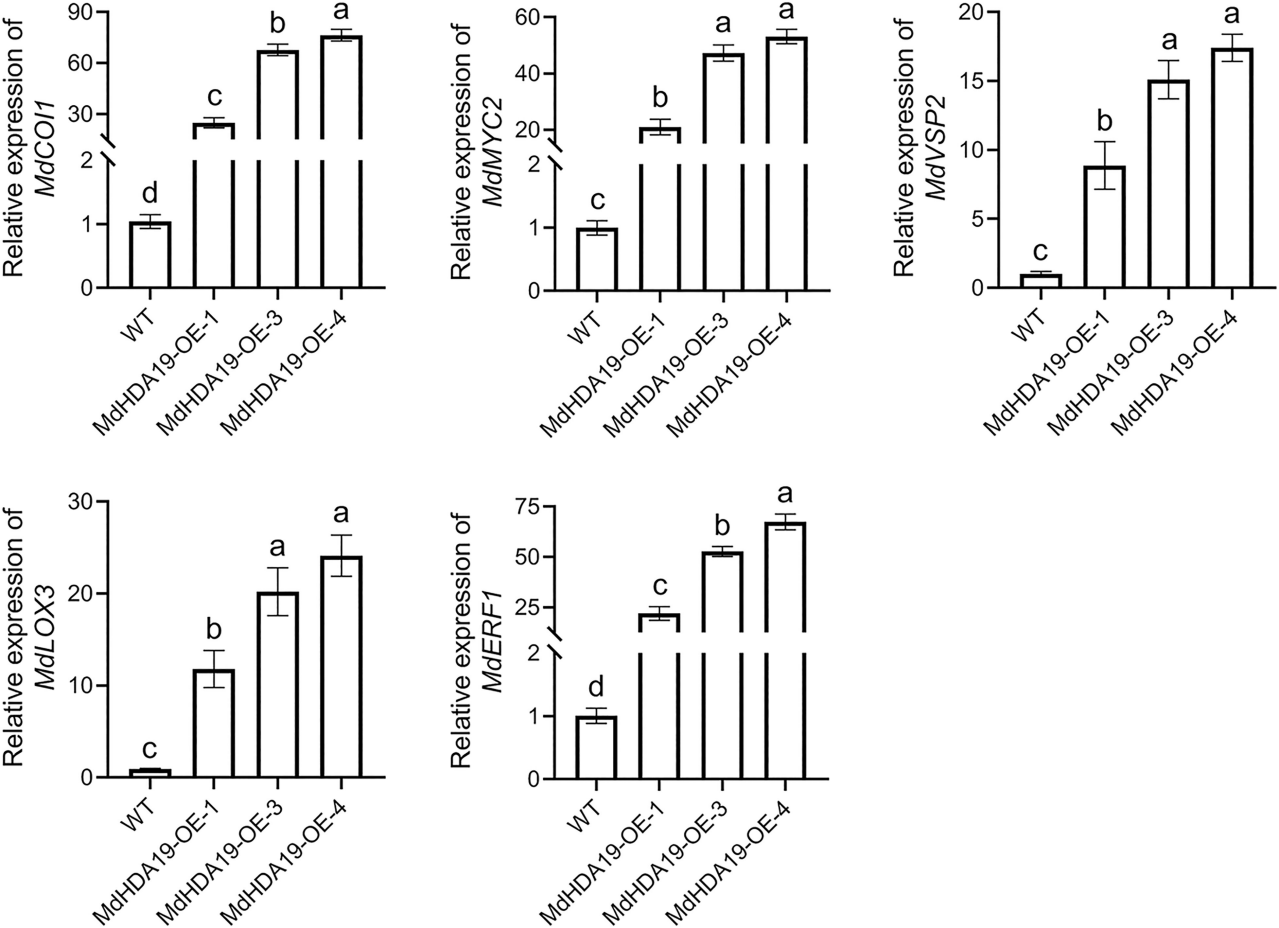
Relative expression of genes related to the jasmonic acid (JA) and ethylene (ET) signalling pathways. Bars with different letters are significantly different at *p* < 0.05 according to one‐way analysis of variance (Tukey's test). Data are shown as mean ± *SD*.

Subsequently, stable transgenic apple calli expressing *MdHDA19* were established to investigate the function of *MdHDA19* (Figure 4a). The calli were infected with *V. mali* for 3 days and H_2_O_2_ and O^2−^ levels were measured at 3 days post‐inoculation (dpi). The results indicated that *MdHDA19*‐OE‐1/3/4 apple calli exhibited increased resistance to *V. mali* infection, with 30.4% ± 7.5%, 51.1% ± 1.5%, and 60.7% ± 4.7% reductions in lesion areas compared to the WT (Figure 4b,c). In addition, *MdHDA19‐*overexpressing apple calli exhibited significantly higher levels of ROS compared to the WT. The H_2_O_2_ content and O^2−^ content of *MdHDA19*‐OE‐1/3/4 were 1.39 ± 0.11, 1.56 ± 0.02, and 1.78 ± 0.14 and 1.71 ± 0.13, 2 ± 0.15, and 2.23 ± 0.2 times higher than the WT, respectively (Figure 4d,e). We then made an association study by showing the *R* ^2^ and *p* values between levels of *MdHDA19* transcripts and lesion area, H_2_O_2_ content, and O^2−^ content (Table S1). These results demonstrate that *MdHDA19* can enhance the resistance of apple calli to *V. mali*. Similarly, we measured the expression levels of ET and JA signal transduction‐related genes in apple calli. Consistent with the results of GL‐3 leaves, they were also upregulated in the *MdHDA19*‐overexpressing apple calli compared with the WT (Figure 5); therefore, we thought that *MdHDA19* was involved in the ET and JA signalling pathways and thus enhanced apple resistance to *V. mali*.

### 2.5 MdVQ12 functions as a positive transcriptional regulator of MdWRKY23 and confers apple resistance to *V. mali* by activating the ET and JA signalling pathways

**FIGURE 6:**
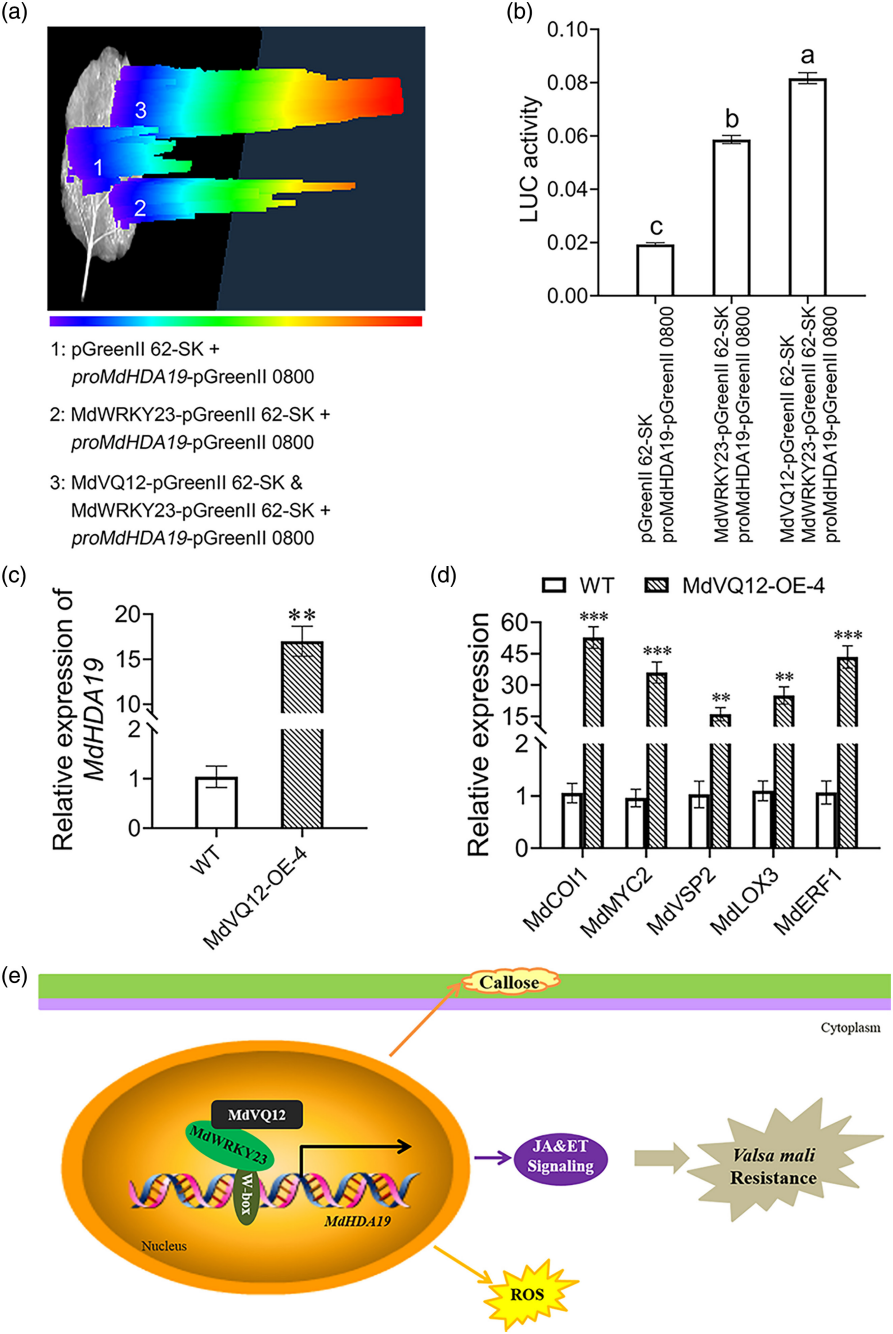
MdVQ12 functions as a positive transcriptional regulator of MdWRKY23. (a) Luciferase (LUC) imaging assay detecting the transcriptional activity of MdWRKY23. (b) LUC activity assay detecting the transcriptional activity of MdWRKY23. (c) Relative expression of *MdHDA19* in wild‐type (WT) and *MdVQ12*‐OE apple leaves. (d) Relative expression of genes related to the jasmonic acid (JA) and ethylene (ET) signalling pathways in WT and *MdVQ12*‐OE apple leaves. (e) Working model showing MdVQ12 confers apple resistance to *Valsa mali* by modulating the expression of *MdHDA19*. Bars with different letters are significantly different at *p* < 0.05 according to one‐way analysis of variance (Tukey's test). **p < 0.01, ***p < 0.001; *t* test. Data are shown as mean ± *SD*.

Because VQ proteins usually act in conjunction with TFs to affect the transcriptional activity of TFs (Jing & Lin, 2015; Lei et al., 2017), we investigated *MdVQ12*'s influence on the transcriptional activity of MdWRKY23 by detecting luminescence signals and LUC activity. *N. benthamiana* leaves were co‐infiltrated with various combinations: combination 1, combination 2, and combination 2 together with MdVQ12‐pGreenII 62‐SK (combination 5). The results showed that both luminescence signal and LUC activity were stronger when combination 5 was co‐expressed than when combination 2 was co‐expressed (Figure 6a,b), indicating that MdVQ12 functions as a positive transcriptional regulator of MdWRKY23.

As MdWRKY23 was identified to transcriptionally activate *MdHDA19*, and MdVQ12 acts as a positive transcriptional regulator of MdWRKY23, we assessed the relative expression levels of *MdHDA19* in *MdVQ12*‐overexpressing lines. The results indicated a significant upregulation of *MdHDA19* (Figure 6c). We postulated that *MdVQ12* may also modulate the ET and JA signalling pathways mediated by *MdHDA19*. Accordingly, we quantitatively assessed genes associated with the ET and JA signalling pathways. The results showed a significant increase in their accumulation in *MdVQ12*‐overexpressing lines (Figure 6d), suggesting that *MdVQ12* enhances apple resistance to *V. mali* by regulating *MdHDA19* expression and thereby activating the ET and JA signalling pathways.

Taken together, *MdVQ12* was able to activate *MdHDA19*‐mediated ET and JA signalling pathways by enhancing the transcriptional activation activity of MdWRKY23 on *MdHDA19*, which in turn further enhanced the resistance against *V. mali*.

## 3 DISCUSSION

Apple Valsa canker, attributed to *V. mali*, inflicts significant economic losses. The poor efficiency and effectiveness of traditional control methods have limited apple production. Identifying disease resistance genes remains the most cost‐effective disease control approach. VQ proteins regulate many aspects of plant growth and development, including plant disease resistance, by interacting with TFs, modulating their transcriptional activity (Chi et al., 2013; Jing & Lin, 2015; Lai et al., 2011; Li et al., 2014). However, the molecular mechanisms of VQ proteins in *V. mali* resistance remain unclear. In this study, we found that *MdVQ12* confers apple resistance to *V. mali* by facilitating MdWRKY23's transcriptional activation of *MdHDA19* in the ET and JA signalling pathways.

Previous studies have shown that the function of VQ proteins is largely affected by the VQ motif (FxxhVQxhTG) (Jing & Lin, 2015). There is a conserved VQ motif in MdVQ12; therefore, MdVQ12 should putatively have the typical functions of VQ proteins. VQ proteins regulate plant immunity, for example, *AtVQ21* overexpression in *Arabidopsis* enhanced resistance to *P. syringae* (Andreasson et al., 2005) but reduced resistance to *B. cinerea* (Fiil & Petersen, 2011; Petersen et al., 2010). In our study, *MdVQ12* increased apple resistance to *V. mali*, contributing to VQ protein research in plant immunity. VQ proteins function by recruiting other TFs (i.e., MdWRKY23) that activate downstream genes involved in disease resistance (Lai et al., 2011; current study). Here, we found that MdVQ12 could interact with MdWRKY23.

The WRKY domain in WRKY TFs is required for protein–protein interactions (Eulgem et al., 2000). Here, we established that the WRKY domain in MdWRKY23 is essential for the MdWRKY23–MdVQ12 interaction. The *cis*‐acting element W‐box ([C/T] TGAC [C/T]) in gene promoters serves as a binding site for WRKY TFs, and mutations within TGAC can impair binding (Rushton et al., 2010). Our DAP‐seq results revealed the presence of a W‐box in the promoter of the histone deacetylase gene *MdHDA19*, and MdWRKY23 was found to bind to it, thereby activating *MdHDA19* expression. Previous studies have demonstrated that HDACs exhibit histone deacetylase activity attributed to their role as histone deacetylases, and this activity is closely associated with ET and JA signalling. For instance, *Arabidopsis* HDA6 displays histone deacetylase activity, which can globally influence histone acetylation levels. *HDA6* mutant and RNAi plants exhibit higher levels of histone acetylation compared to the WT, and the expression of ET and JA signalling‐related genes is downregulated (Wu et al., 2008). Similarly, *Arabidopsis* HDA19 also possesses histone deacetylase activity. Overexpression of *HDA19* leads to reduced histone acetylation levels compared to the WT, enhanced resistance to the fungal pathogen *Alternaria*, and upregulation of ET and JA signalling‐related genes. Conversely, *HDA19* RNAi plants show increased histone acetylation levels and downregulated expression of ET and JA signalling‐related genes (Zhou et al., 2005). Therefore, we propose that MdHDA19 has histone deacetylase activity and its activation is associated with both ET and JA signalling pathways. In this study, we found that several ET and JA signalling‐related genes were upregulated in *MdHDA19*‐overexpressing apple leaves and calli, leading to increased resistance to the fungal pathogen *V. mali*. Additionally, these genes exhibited significantly decreased expression levels after TSA treatment. These results indicate that MdHDA19 has histone deacetylase activity and that the activation of this activity is related to the ET and JA signalling pathways, ultimately enhancing apple resistance to the pathogenic fungus *V. mali*. Moreover, consistent with previous reports that VQ proteins can modulate the transcriptional activity of TFs, we demonstrated that MdVQ12 could promote the transcriptional activation of MdWRKY23 on *MdHDA19*. However, there are no reports on the relationship between VQ proteins and HDACs. Therefore, this study offers novel insights into the regulatory mechanism of VQ proteins.

In plant defence, ROS burst and callose deposition are fundamental processes crucial for the defensive reaction (Boller & Felix, 2009; Schwessinger & Ronald, 2012). Callose deposition increases cell wall thickness, thereby slowing pathogen invasion, making it a universal model for quantifying plant defence responses (Luna et al., 2011; Nishimura et al., 2003). ROS play a central role in defending against pathogen invasion and activating plant immune responses, conferring resistance (Chen et al., 1993; Kariola et al., 2005; Qi et al., 2017; Sharma et al., 2012). VQ proteins, functioning as transcriptional regulators, typically co‐regulate plant immune responses along with their interacting TFs, including ROS burst and callose deposition. In this study, we confirmed that *MdVQ12* was induced during *V. mali* infection and significantly enhanced ROS and callose accumulation, advancing our understanding of VQ protein‐mediated disease resistance.

The results of our regulatory approach are summarized in a model in Figure 6e. MdVQ12 interacts with MdWRKY23 to form a complex in this model. This interaction modulates the transcriptional capacity of MdWRKY23 towards *MdHDA19*, and they are novel components of the regulatory network of apple Valsa canker resistance. Furthermore, *MdHDA19* contributes to apple resistance against *V. mali* by participating in the JA and ET signalling pathways. In conclusion, our study establishes that *MdVQ12* acts as an activator within the MdWRKY23‐MdHDA19 module, which mediates apple Valsa canker resistance. This work provides a novel regulatory network for understanding disease modulation by VQ proteins. Our findings offer theoretical guidance and technical support for the cultivation of disease‐resistant germplasm resources.

## 4 EXPERIMENTAL PROCEDURES

### 4.1 Plant and microbe materials

The apple tissue culture seedlings are of the GL‐3 genotype (Dai et al., 2013). The GL‐3 plantlets and apple cv. Orin calli were cultured on Murashige and Skoog (MS) medium at 25°C.

*N. benthamiana* seedlings were grown in a growth chamber and the *V. mali* WT strain 03‐8 (Yin et al., 2015) were grown in incubators at 25°C.

### 4.2 RT‐qPCR analysis

Quick RNA isolation kits (Huayueyang Biotechnology) and RevertAid First Strand cDNA synthesis kits (Thermo Scientific) were used for total RNA extraction and cDNA synthesis, respectively. The LightCycler 96 System (Roche) and RealStar Green Mixture (GenStar) were used for RT‐qPCR assays. The reaction procedures were as follows: 10 min at 95°C; then 40 cycles of 15 s at 95°C, 30 s at 60°C, and 30 s at 72°C. *MdMDH* (Perini et al., 2014) was used as an internal control for the normalization of gene expression. To analyse the results, the 2^−ΔΔ*C*t^ method (Livak & Schmittgen, 2001) was performed. The primers that we used were listed in Table S3. All operations were performed in triplicate.

### 4.3 Bioinformatic analysis, molecular cloning, and vector construction

The NCBI (National Center for Biotechnology Information) website (https://www.ncbi.nlm.nih.gov/) was used for domain analysis. The Protter website (http://wlab.ethz.ch/protter/start/) was used for the visualization of protein features. The Phyre2 website (http://www.sbg.bio.ic.ac.uk/phyre2/html/page.cgi?id=index) was used for the three‐dimensional structural prediction.

The specific primers for molecular cloning were designed based on the sequences from the GDR (Genome Database for Rosaceae) website (https://www.rosaceae.org/) and listed in Table S4. The genes were amplified from the GL‐3 tissue culture seedling cDNA using the Phanta Max super‐fidelity DNA polymerase (Vazyme).

The recombinant vector for stable overexpression (*MdVQ12*‐OE, *MdHDA19*‐OE) was constructed using pK2GW7 combined with the hemagglutunin (HA) tag. The recombinant vectors for stable silencing (*MdVQ12*‐RNAi) and transient silencing (*MdWRKY23*‐RNAi‐GFP, *MdHDA19*‐RNAi) were constructed using pK7GWIWG2D (II) containing the green fluorescent protein (GFP) tag. The pK2GW7 and pK7GWIWG2D (II) vectors were provided by Qingmei Guan of Northwest A&F University. The recombinant vectors for subcellular localization (*MdVQ12*‐GFP) and transient overexpression (*MdVQ12*‐OE‐GFP, *MdHDA19*‐OE) were constructed using pCAMBIA1302 containing the GFP tag, which was provided by Xiaojie Wang of Northwest A&F University. The recombinant vectors for BiFC (MdVQ12‐YFP^C^, MdWRKY23‐YFP^N^) were constructed using pSPYNE‐35S and pSPYCE‐35S. The recombinant vectors for GUS activity detection (MdWRKY23‐pGreenII 62‐SK, *proMdHDA19*‐pCB308) were constructed using pGreenII 62‐SK and pCB308. The recombinant vectors for luminescence signal and LUC activity detection (MdVQ12‐pGreenII 62‐SK, MdWRKY23‐pGreenII 62‐SK, p*roMdHDA19*‐pGreenII 0800) were constructed using pGreenII 62‐SK and pGreenII 0800. The recombinant vectors for Y2H (MdWRKY23‐AD, MdVQ12‐BD) were constructed using pGADT7 and pGBKT7. The recombinant vectors for Y1H (MdWRKY23‐AD, *proMdHDA19*‐pHIS2) were constructed using pGADT7 and pHIS2. The recombinant vector for EMSA (MdWRKY23‐His) was constructed using pET‐28a.

### 4.4 Genetic transformation, subcellular localization, and BiFC

The *Agrobacterium tumefaciens* strains EHA105 and GV3101 (pSoup‐P19) were used for stable and transient expression, respectively. Transient expression assays were performed using the leaves of 4‐week‐old GL‐3 tissue culture seedlings and *N. benthamiana* seedlings. Experiments were performed according to Zhang et al. (2018) and Sun et al. (2018), respectively. Stable expression assays were performed using 15‐day‐old Orin apple calli and the leaves of 4‐week‐old GL‐3 tissue culture seedlings. Experiments were performed according to Xie et al. (2012) and Wang et al. (2017), respectively.

For the subcellular localization and BiFC, after transient expression in the leaves of *N. benthamiana* seedlings for 48 h, the fluorescence signal observation was performed using an FV3000 laser scanning confocal microscope (LSCM) (Olympus). Experiments were performed three times.

### 4.5 LUC and GUS analysis

After transient expression in Orin apple calli and the leaves of *N. benthamiana* seedlings for 48 h, the luminescence signal observation was performed using d‐luciferin (Solarbio) and the PlantView100 multispectral dynamic fluorescence microscopic imaging system (Biolight Biotechnology); the LUC activity was measured using Dual Luciferase Reporter Gene Assay Kits (Yeasen Biotechnology); the GUS staining assays were performed using GUS stain Kits (Coolaber); the GUS activity was measured using GUS gene quantitative detection kits (Coolaber). Tests were performed at least three times.

### 4.6 Pathogen infection, ROS and callose contents detection, and TSA treatment

Ten‐day‐old Orin apple calli, the leaves of 5‐week‐old, and twigs of 4‐month‐old GL‐3 seedlings were infected with *V. mali* following the protocol of Han et al. (2022). They were analysed at 3 or 4 dpi, 32 or 36 hpi, and 48 hpi, respectively. The statistics of lesion areas and lengths were performed using the ImageJ software. The H_2_O_2_ content, O^2−^ content and production rate, and callose content were detected using hydrogen peroxide assay kits, superoxide anion assay kits (spectrophotometry), and callose assay kits (fluorescence) (Comin Biotechnology), respectively. TSA (Solarbio) was used to inhibit histone deacetylation activity, and the TSA treatment assay was performed according to Mehdi et al. (2016). The experiments were performed with three replications.

### 4.7 IP‐MS, Co‐IP, and Y2H

The leaves of *MdVQ12*‐overexpressing GL‐3 tissue culture seedlings and transiently expressed *N. benthamiana* seedlings were used for total protein extraction. Pierce anti‐HA magnetic beads (Thermo Scientific) and GFP‐trap A beads (Chromotek) were used to separate fusion proteins for IP‐MS and Co‐IP, respectively. IP‐MS was conducted using the Q Exactive HF‐X ultrahigh‐resolution liquid chromatography‐mass spectrometry (Thermo Scientific). Western blotting was performed for Co‐IP assays following the description of Nie et al. (2021). Y2H assays were performed using the yeast strain Y2H Gold. The experiments were conducted as described by Han et al. (2019). All experiments were repeated three times.

### 4.8 DAP‐seq, EMSA, and Y1H

Candidate target genes of MdWRKY23 were obtained via DAP‐seq, which was conducted by the Genedenovo Biotechnology Co. Ltd. (Guangzhou, China).

The fusion protein MdWRKY23‐His was purified using the Ni‐NTA resin (Thermo Scientific) as described by Nie et al. (2021). EMSA was performed using chemiluminescent EMSA kits (Beyotime Biotechnology) as described by Wang et al. (2018). Y1H assays were performed using the yeast strain Y187. The experiments were conducted as described by Yu et al. (2020). 3‐amino‐1,2,4‐triazole (Coolaber) was used for self‐activation inhibition. The experiment was repeated three times.

### 4.9 Statistical analysis

All experiments were replicated at least three times. The one‐way analysis of variance (ANOVA, Tukey's test) or *t* test analysis of GraphPad Prism v. 8.0.2 was used to determine statistical significance. Data are shown as mean ± *SD*.

## CONFLICT OF INTEREST STATEMENT

The authors declare no conflicts of interest.

## Supporting information


**FIGURE S1.** Bioinformatic analysis of MdVQ12.

**FIGURE S2.** *MdVQ12* positively regulates apple calli resistance to *Valsa mali*.

**FIGURE S3.** Identification of stable overexpression apple tissue culture seedlings of *MdVQ12*.

**FIGURE S4.** Identification of stable gene silencing apple tissue culture seedlings of *MdVQ12*.

**FIGURE S5.** *MdVQ12*‐induced resistance is *MdWRKY23*‐dependent.

**FIGURE S6.** *MdHDA19* confers apple resistance to *Valsa mali*.

**FIGURE S7.** Relative expression of genes related to the jasmonic acid (JA) and ethylene (ET) signalling pathways.

**FIGURE S8.** Relative expression of *MdHDA19* and genes related to the jasmonic acid (JA) and ethylene (ET) signalling pathways.

**TABLE S1.** Association study of *R* ^2^ and *p* values between levels of gene transcripts and each trait.

**TABLE S2.** The WRKY domain together with N‐terminal segment of MdWRKY23 can interact with MdVQ12.

**TABLE S3.** Primers used in this study for reverse transcription‐quantitative PCR assays.

**TABLE S4.** Primers used in this study for molecular cloning.

## Data Availability

The data that support the findings of this study are available from the corresponding author upon reasonable request.
