# Supplementary material for: MdVQ12 confers resistance to Valsa mali by regulating MdHDA19 expression in apple
Source: Mol Plant Pathol. 2023 Dec 10;25(1):e13411. doi: 10.1111/mpp.13411 (PMC10788466; doi:10.1111/mpp.13411)
Supplement: Supplementary file 1 — FIGURE S1. Bioinformatic analysis of MdVQ12. [file MPP-25-e13411-s005.docx]

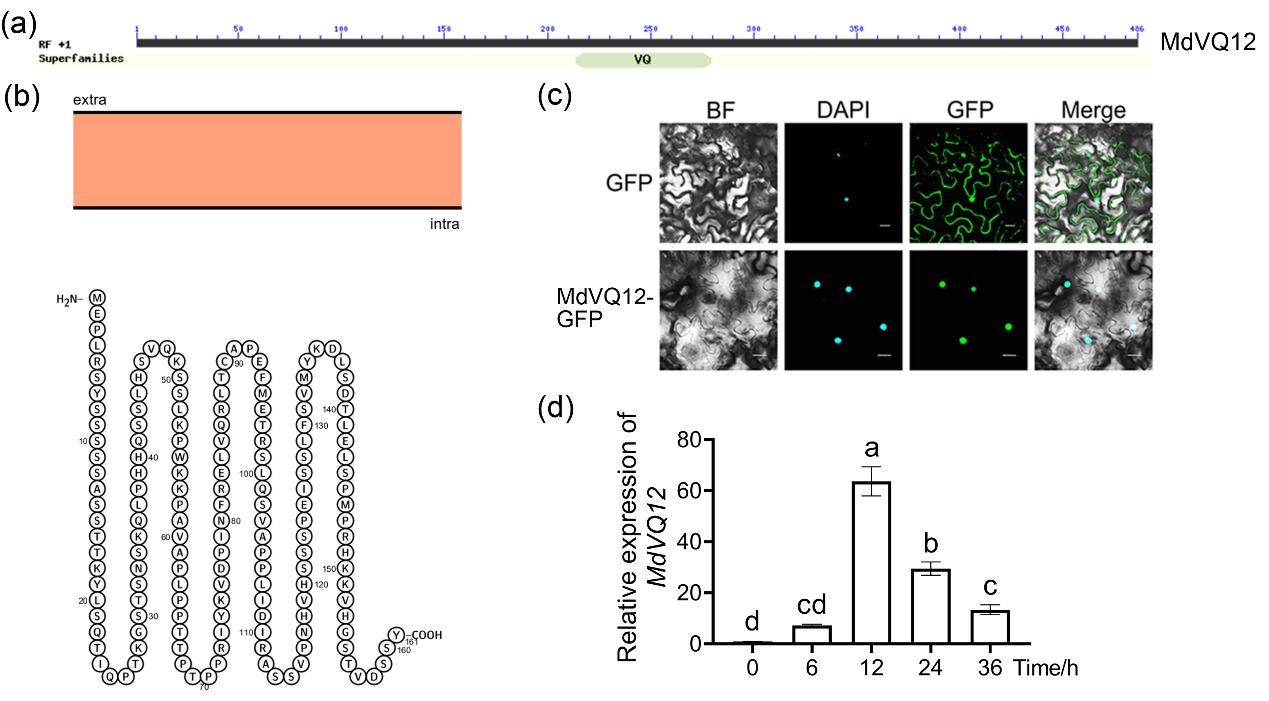


**FIGURE S1** Bioinformatic analysis of MdVQ12. (a) Domain analysis of MdVQ12. (b) Visualization of protein feature of MdVQ12. (c) Subcellular localization of MdVQ12. Bars = 20 μm. (d) Relative expression of *MdVQ12* during *V. mali* infection. Bars with different letters are significantly different at *P*<0.05 according to one-way ANOVA (Tukey’s test). Data are shown as mean ± SD.
