# Supplementary material for: MdVQ12 confers resistance to Valsa mali by regulating MdHDA19 expression in apple
Source: Mol Plant Pathol. 2023 Dec 10;25(1):e13411. doi: 10.1111/mpp.13411 (PMC10788466; doi:10.1111/mpp.13411)
Supplement: Supplementary file 2 — FIGURE S2. MdVQ12 positively regulates apple calli resistance to Valsa mali. [file MPP-25-e13411-s001.docx]

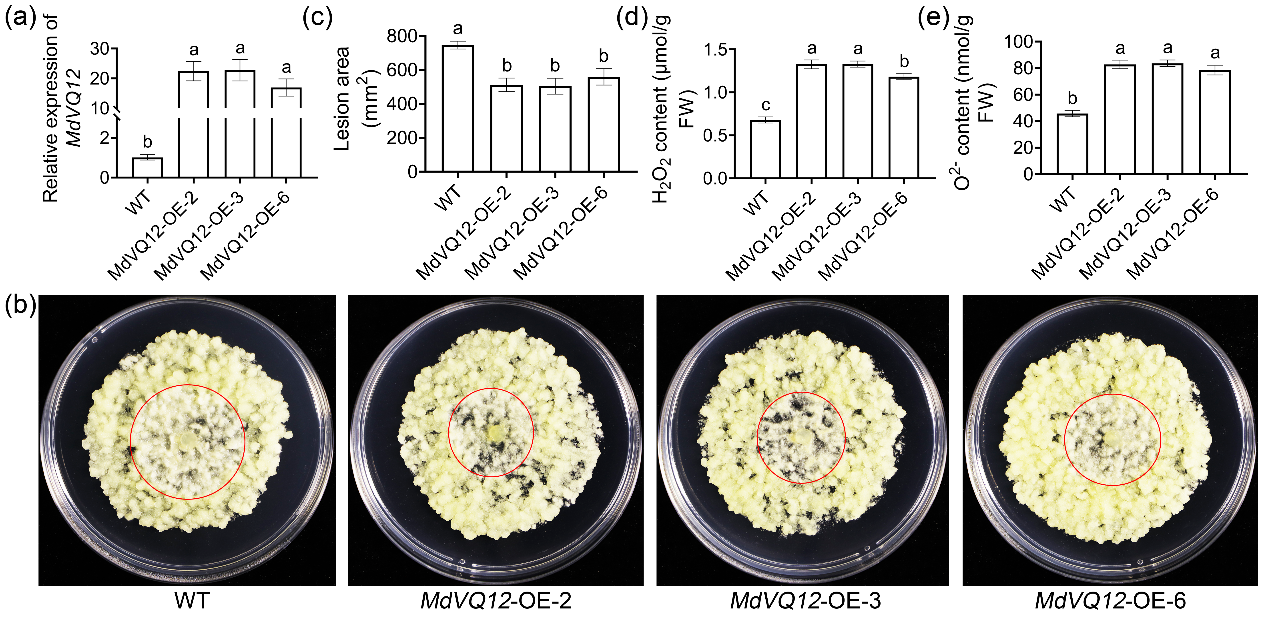


**FIGURE S2** *MdVQ12* positively regulates apple calli resistance to *V. mali*. (a) Relative expression of *MdVQ12*. (b) Disease symptoms of WT, *MdVQ12*-OE-2, *MdVQ12*-OE-3, and *MdVQ12*-OE-6 apple calli at 4 dpi. (c) Lesion areas of WT, *MdVQ12*-OE-2, *MdVQ12*-OE-3, and *MdVQ12*-OE-6 apple calli at 4 dpi. (d) H_2_O_2_ contents of WT, *MdVQ12*-OE-2, *MdVQ12*-OE-3, and *MdVQ12*-OE-6 apple calli at 4 dpi. (e) O^2-^ contents of WT, *MdVQ12*-OE-2, *MdVQ12*-OE-3, and *MdVQ12*-OE-6 apple calli at 4 dpi. Bars with different letters are significantly different at *P*<0.05 according to one-way ANOVA (Tukey’s test). Data are shown as mean ± SD.
