# Supplementary material for: MdVQ12 confers resistance to Valsa mali by regulating MdHDA19 expression in apple
Source: Mol Plant Pathol. 2023 Dec 10;25(1):e13411. doi: 10.1111/mpp.13411 (PMC10788466; doi:10.1111/mpp.13411)
Supplement: Supplementary file 3 — FIGURE S3. Identification of stable overexpression apple tissue culture seedlings of MdVQ12. [file MPP-25-e13411-s004.docx]

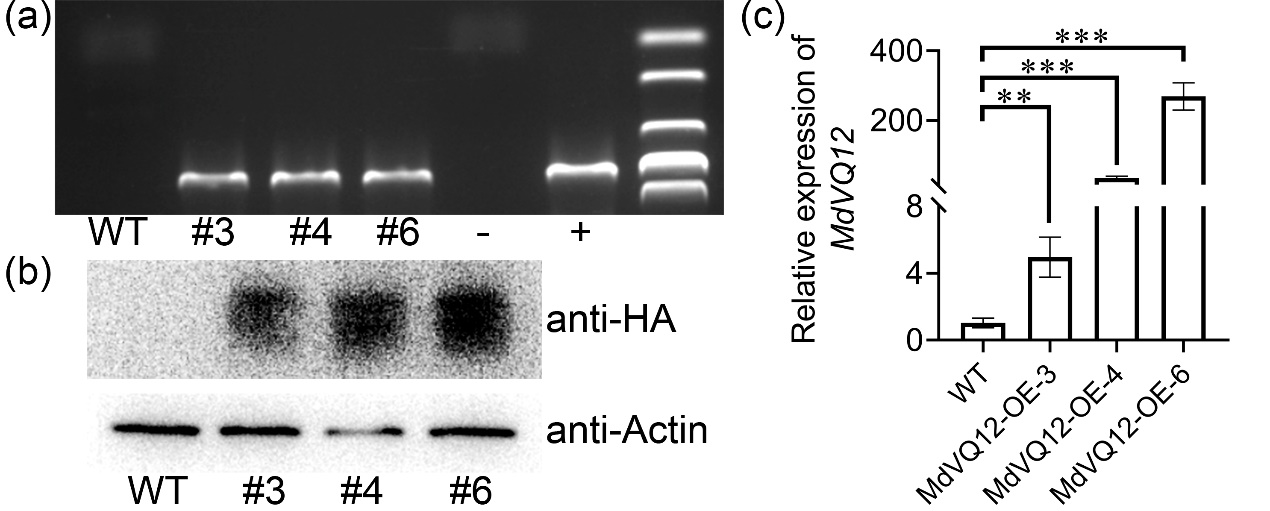


**FIGURE S3** Identification of stable overexpression apple tissue culture seedlings of *MdVQ12*. (a) Identification of *MdVQ12*-OE transgenic apple tissue culture seedlings by DNA detection. (b) Identification of *MdVQ12*-OE transgenic apple tissue culture seedlings by western blot. (c) Relative expression of *MdVQ12* of wild type and OE lines. **, *P* < 0.01; ***, *P* < 0.001; *t*-test. Data are shown as mean ± SD.
