# Supplementary material for: MdVQ12 confers resistance to Valsa mali by regulating MdHDA19 expression in apple
Source: Mol Plant Pathol. 2023 Dec 10;25(1):e13411. doi: 10.1111/mpp.13411 (PMC10788466; doi:10.1111/mpp.13411)
Supplement: Supplementary file 4 — FIGURE S4. Identification of stable gene silencing apple tissue culture seedlings of MdVQ12. [file MPP-25-e13411-s008.docx]

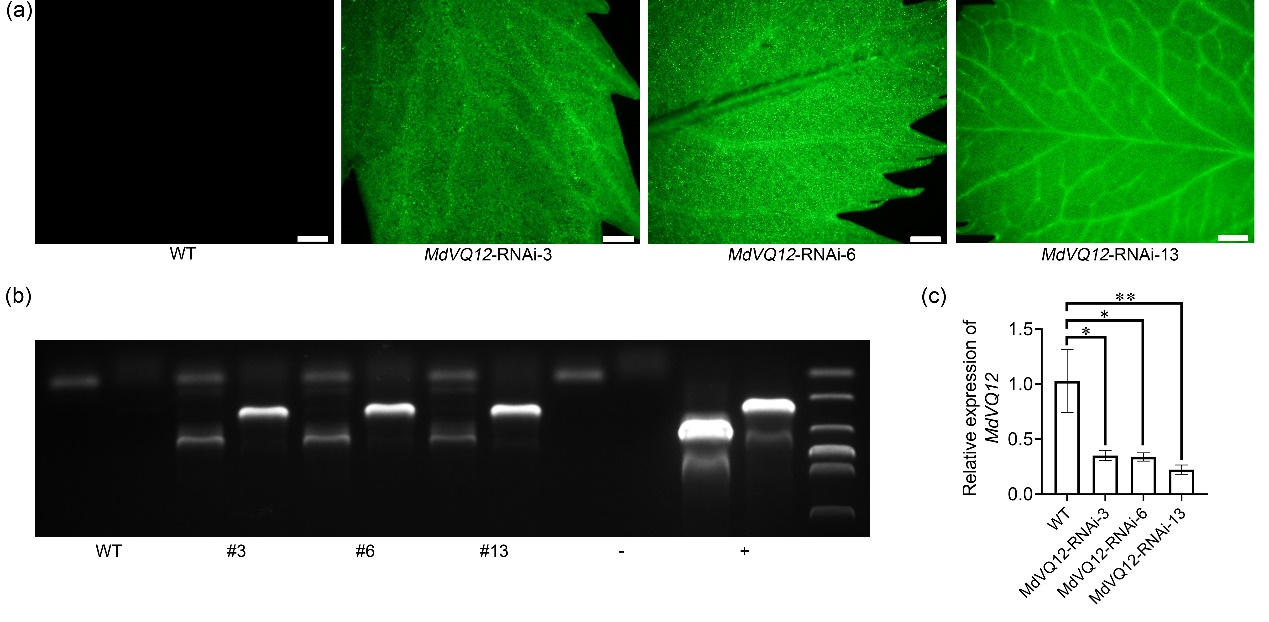


**FIGURE S4** Identification of stable gene silencing apple tissue culture seedlings of *MdVQ12*. (a) Identification of *MdVQ12*-RNAi transgenic apple tissue culture seedlings by fluorescent labeling. Bars = 200 μm. (b) Identification of *MdVQ12*-RNAi transgenic apple tissue culture seedlings by DNA detection. The gene silencing vector contains two DNA fragments, both of which must be simultaneously detected to confirm the successful acquisition of RNAi genetically transformed material. Therefore, the detection results for each pair of lanes represent a single *MdVQ12*-RNAi line. (c) Relative expression of *MdVQ12* of wild type and RNAi lines. *, *P* < 0.05; **, *P* < 0.01; *t*-test. Data are shown as mean ± SD.
