# Supplementary material for: MdVQ12 confers resistance to Valsa mali by regulating MdHDA19 expression in apple
Source: Mol Plant Pathol. 2023 Dec 10;25(1):e13411. doi: 10.1111/mpp.13411 (PMC10788466; doi:10.1111/mpp.13411)
Supplement: Supplementary file 5 — FIGURE S5. MdVQ12‐induced resistance is MdWRKY23‐dependent. [file MPP-25-e13411-s011.docx]

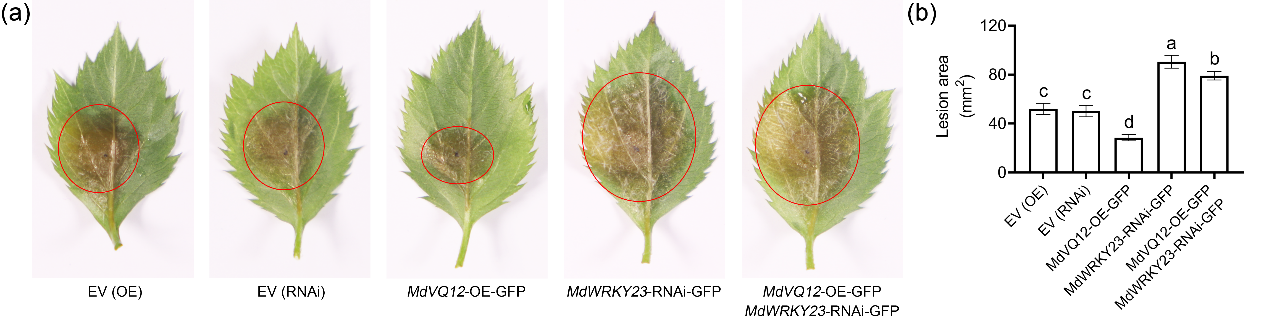


**FIGURE S5** *MdVQ12*-induced resistance is *MdWRKY23*-dependent. (a) Disease symptoms of EV (OE), EV (RNAi), *MdVQ12*-OE-GFP, *MdWRKY23*-RNAi-GFP, and *MdVQ12*-OE-GFP&*MdWRKY23*-RNAi-GFP apple leaves at 36 hpi. (b) Lesion areas of EV (OE), EV (RNAi), *MdVQ12*-OE-GFP, *MdWRKY23*-RNAi-GFP, and *MdVQ12*-OE-GFP&*MdWRKY23*-RNAi-GFP apple leaves at 36 hpi. Bars with different letters are significantly different at *P*<0.05 according to one-way ANOVA (Tukey’s test). Data are shown as mean ± SD.
