# Supplementary material for: MdVQ12 confers resistance to Valsa mali by regulating MdHDA19 expression in apple
Source: Mol Plant Pathol. 2023 Dec 10;25(1):e13411. doi: 10.1111/mpp.13411 (PMC10788466; doi:10.1111/mpp.13411)
Supplement: Supplementary file 6 — FIGURE S6. MdHDA19 confers apple resistance to Valsa mali. [file MPP-25-e13411-s006.docx]

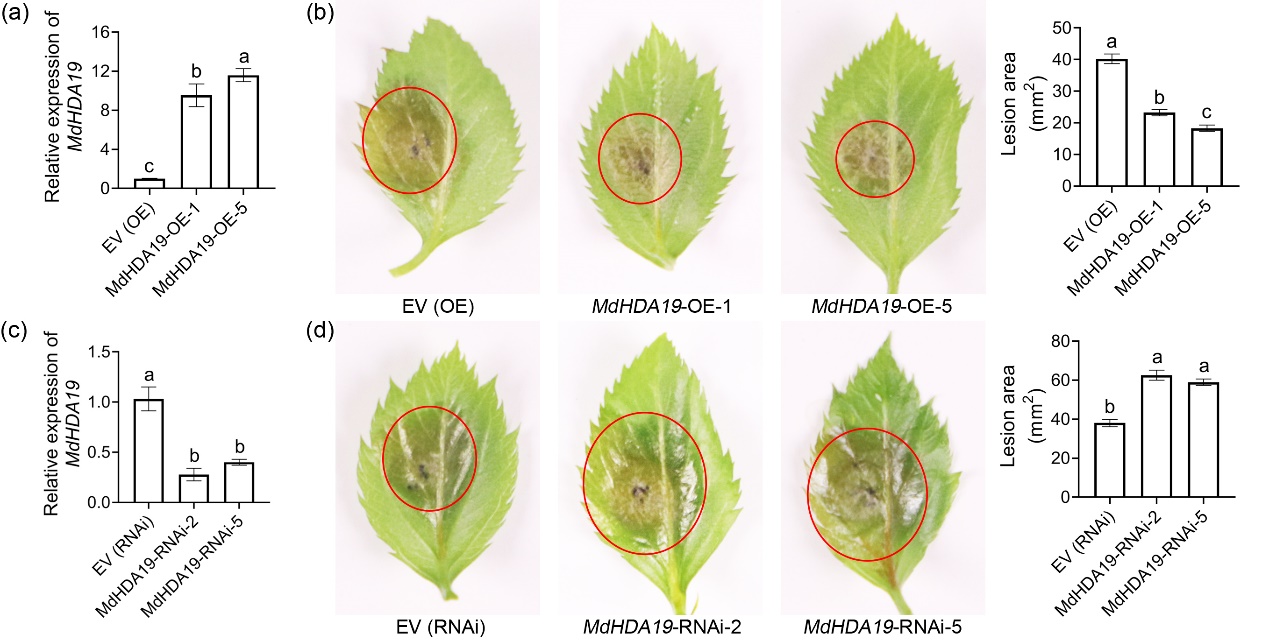


**FIGURE S6** *MdHDA19* confers apple resistance to *V. mali*. (a) Relative expression of *MdHDA19*. (b) Lesion areas of EV (OE), *MdHDA19*-OE-1, and *MdHDA19*-OE-5 apple leaves at 32 hpi. (c) Relative expression of *MdHDA19*. **(**d) Lesion areas of EV (RNAi), *MdHDA19*- RNAi-2, and *MdHDA19*-RNAi-5 apple leaves at 32 hpi. Bars with different letters are significantly different at *P*<0.05 according to one-way ANOVA (Tukey’s test). Data are shown as mean ± SD.
