# Supplementary material for: MdVQ12 confers resistance to Valsa mali by regulating MdHDA19 expression in apple
Source: Mol Plant Pathol. 2023 Dec 10;25(1):e13411. doi: 10.1111/mpp.13411 (PMC10788466; doi:10.1111/mpp.13411)
Supplement: Supplementary file 7 — FIGURE S7. Relative expression of genes related to the jasmonic acid (JA) and ethylene (ET) signalling pathways. [file MPP-25-e13411-s003.docx]

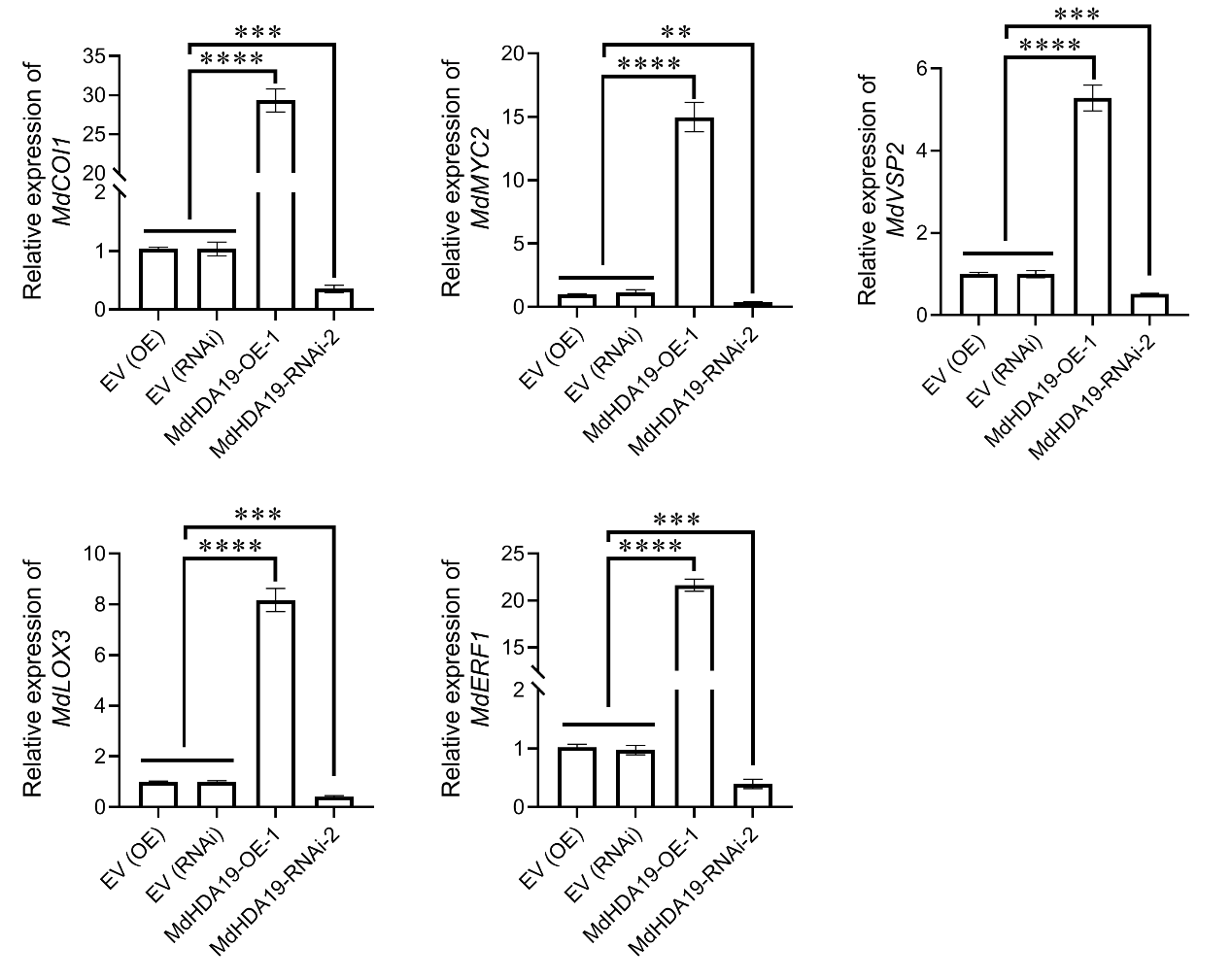


**FIGURE S7** Relative expression of genes related to the JA and ET signaling pathways. **, *P* < 0.01; ***, *P* < 0.001; ****, *P* < 0.0001; *t*-test. Data are shown as mean ± SD.
