# Supplementary material for: MdVQ12 confers resistance to Valsa mali by regulating MdHDA19 expression in apple
Source: Mol Plant Pathol. 2023 Dec 10;25(1):e13411. doi: 10.1111/mpp.13411 (PMC10788466; doi:10.1111/mpp.13411)
Supplement: Supplementary file 8 — FIGURE S8. Relative expression of MdHDA19 and genes related to the jasmonic acid (JA) and ethylene (ET) signalling pathways. [file MPP-25-e13411-s009.docx]

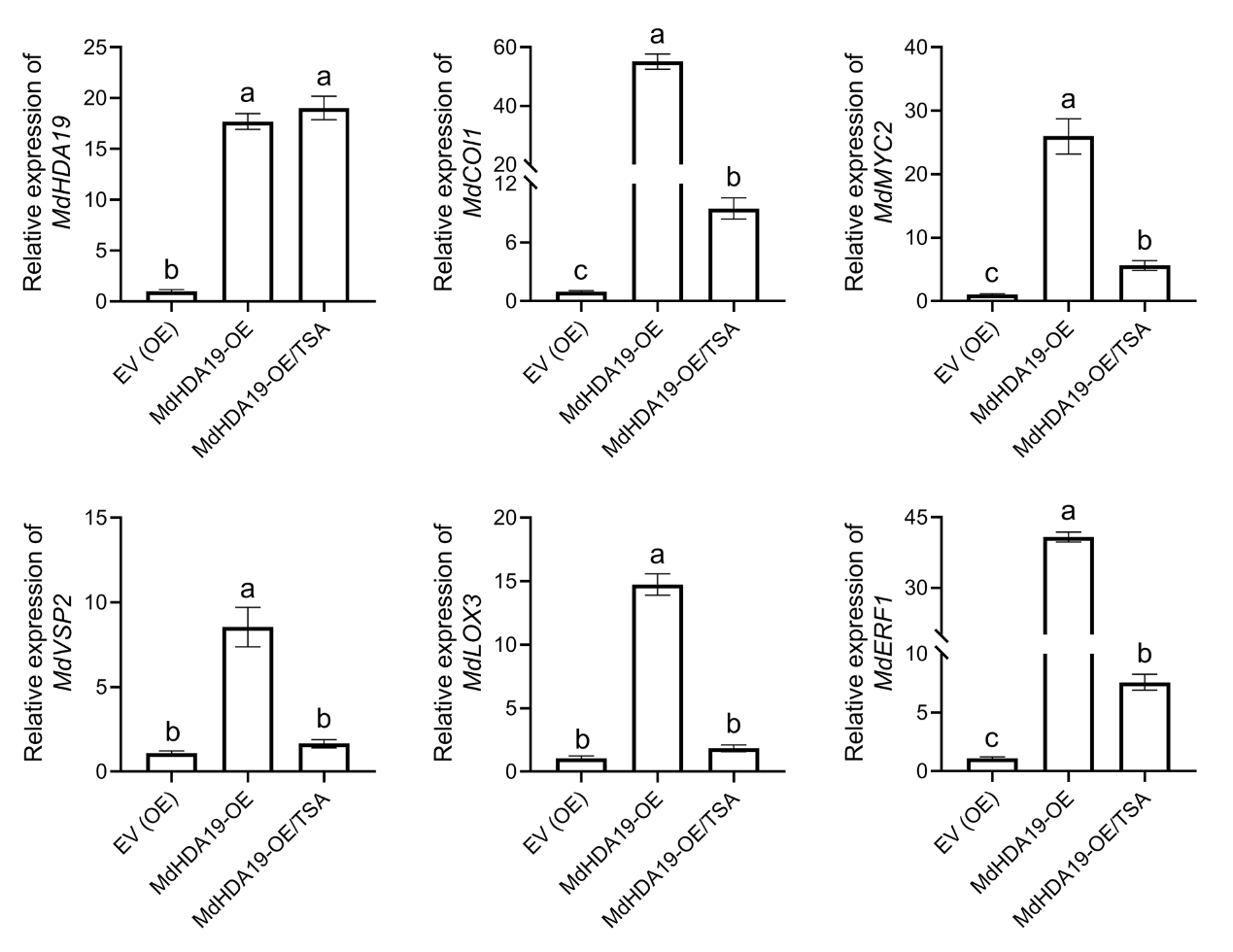


**FIGURE S8** Relative expression of *MdHDA19* and genes related to the JA and ET signaling pathways. Bars with different letters are significantly different at *P*<0.05 according to one-way ANOVA (Tukey’s test). Data are shown as mean ± SD.
