# Supplementary material for: MdVQ12 confers resistance to Valsa mali by regulating MdHDA19 expression in apple
Source: Mol Plant Pathol. 2023 Dec 10;25(1):e13411. doi: 10.1111/mpp.13411 (PMC10788466; doi:10.1111/mpp.13411)
Supplement: Supplementary file 10 — TABLE S2. The WRKY domain together with N‐terminal segment of MdWRKY23 can interact with MdVQ12. [file MPP-25-e13411-s010.docx]

**TABLE S2** The WRKY domain together with the N-terminal segment of MdWRKY23 can interact with MdVQ12

| Length of MdWRKY23-AD | MdVQ12-BD | Interaction |
| --- | --- | --- |
| 1-1056 bp |  | √ |
| 1-519 bp |  | × |
| 520-693 bp |  | × |
| 694-1056 bp |  | × |
| 520-1056 bp |  | × |
| 1-693 bp |  | √ |
| 1-606 bp |  | × |
| 607-1056 bp |  | × |

Note: The full length of MdWRKY23 is 1056 bp; the position of the WRKY domain of MdWRKY23 is 520-693 bp.
