# Supplementary material for: MdVQ12 confers resistance to Valsa mali by regulating MdHDA19 expression in apple
Source: Mol Plant Pathol. 2023 Dec 10;25(1):e13411. doi: 10.1111/mpp.13411 (PMC10788466; doi:10.1111/mpp.13411)
Supplement: Supplementary file 11 — TABLE S3. Primers used in this study for reverse transcription‐quantitative PCR assays. [file MPP-25-e13411-s007.docx]

**TABLE S3** Primers used in this study for qRT-PCR assays

| Primer | Primer sequence (5’→3’) |
| --- | --- |
| *MdMDH*-F | CGTGATTGGGTACTTGGAAC |
| *MdMDH*-R | TGGCAAGTGACTGGGAATGA |
| *MdVQ12*-F | TTCCTCTTCCTCTTCAGCCTCCTC |
| *MdVQ12*-R | CGAGTGGAGTGATGACTGGTGATG |
| *MdHDA19*-F | TGCTTCTAGGTGGTGGTGGCTATAC |
| *MdHDA19*-R | TGCTCCAAGTGCTACTCCTGTCTC |
| *MdCOI1*-F | GAGGGCGGCGATGTTCAATCTG |
| *MdCOI1*-R | ACTTCAAGCGGTGGAACGAGTTG |
| *MdMYC2*-F | CAGGTGGACAATGGTCGTCTCTTTC |
| *MdMYC2*-R | TGTGGTGATGGTTGTTGGGTGATG |
| *MdLOX3*-F | AAGACTACCCGTTTGCTGTTGATGG |
| *MdLOX3*-R | GGAGTTCAGAATCACCTTGGACCAC |
| *MdVSP2*-F | GACATCGACGAGACTCTGCTTTCC |
| *MdVSP2*-R | CTCAGCCAAATCCACCCACTCATC |
| *MdERF1*-F | AGGCTGCTGCTTTGGCTTATGAC |
| *MdERF1*-R | CATACTCCATCTCCACAAGCGACTG |
