# Supplementary material for: MdVQ12 confers resistance to Valsa mali by regulating MdHDA19 expression in apple
Source: Mol Plant Pathol. 2023 Dec 10;25(1):e13411. doi: 10.1111/mpp.13411 (PMC10788466; doi:10.1111/mpp.13411)
Supplement: Supplementary file 12 — TABLE S4. Primers used in this study for molecular cloning. [file MPP-25-e13411-s012.docx]

**TABLE S4** Primers used in this study for molecular cloning

| Primer | Primer sequence (5’→3’) |
| --- | --- |
| *MdVQ12*-F | ATGGAACCATTAAGATCTTATTC |
| *MdVQ12*-R | CTAATAGCTCGAGTCCACCGTG |
| *MdWRKY23*-F | ATGGATGTGAAGAAGGATG |
| *MdWRKY23*-R | CTACTGCTCTTGCTTAAG |
| *MdHDA19*-F | ATGGACACCGGCGGCAAC |
| *MdHDA19*-R | TTATATCTGGTCTGCAGG |
